# Trump’s Zero-tolerance Policy: Would a Political Response to a Humanitarian Crisis Work?

**DOI:** 10.15171/ijhpm.2018.80

**Published:** 2018-08-25

**Authors:** Mohammad Karamouzian

**Affiliations:** ^1^School of Population and Public Health, Faculty of Medicine, University of British Columbia, Vancouver, BC, Canada.; ^2^HIV/STI Surveillance Research Center, and WHO Collaborating Center for HIV Surveillance, Institute for Futures Studies in Health, Kerman University of Medical Sciences, Kerman, Iran.

## Dear Editor,

Trump’s zero-tolerance policy for unauthorized border crossers makes zero sense.^1-3^ Numerous human rights organizations and news outlets have described the policy as “nothing short of torture” or “state-sanctioned child abuse” and called for immediate action from the international community.^3-6^ During its implementation, children were taken away from their parents and kept in prison-like appalling conditions (eg, tents, warehouses, cages). Trump’s administration however, claimed that this policy would act as a “deterrent” for the influx of refugees into the United States. Indeed, some hardline immigration groups have viewed the zero-tolerance policy as an integral part of a strategy for keeping US’s borders safe and argued its effectiveness by assuming that people are “hesitating about coming in because of separation.” Conservatives supportive of the policy have also criticized the immigration laws for what they see as a “loophole that incentivizes families to travel together so that they may be released together”^7,8^; arguments that might be attractive but are hard to back up by any available evidence or previous international experience.^9^ The administration has also provided misleading statistics on the increasing number of smugglers to support their cause while smuggling is not the driving force behind increased rates of asylum seekers and is merely a response to an increasing demand for fleeing unrest and violence.^7,10^ Following an international outrage, Trump signed an executive order on June 20, 2018 to “keep the families together” with no clear insight on how the already separated ~3000 children would reunite with their families.^1,8^ While there is no quick fix to the refugee crisis at the US borders, misunderstanding or misrepresenting the underlying social, economic, and political causes that force refugees to consider fleeing their home country is detrimental to reaching a long-term durable solution to this problem.

## Who Are the People Seeking Asylum in the United States?

Although the current administration in the United States tends to portray their country as a major destination for the refugees and asylum seekers in the world bearing most of the global refugee crisis burden, the United Nations High Commissioner for Refugees (UNHCR) statistics tell a different story and show that out of the 68.5 million displaced people across the world, 85% are hosted by the developing countries — the top five being Turkey, Uganda, Pakistan, Lebanon, and Iran.^11^ Nonetheless, the number of people seeking asylum and crossing the Southern border of the USA has been steadily on the rise. Most people trying to legally or illegally cross the US borders come from the so-called Northern Triangle of Central America which includes Guatemala, Honduras, and El Salvador.^12^ However, this is not a new phenomenon and citizens of these countries have been fleeing political instability, civil wars, gang violence, poverty, and broken economies for over three decades.^13^

## How Is the United States Responsible for the Crisis at its Borders?

Trump’s administration feels no empathy for the families and unaccompanied children pouring across the border and has repeatedly tried to misrepresent the asylum seekers as evil criminals, threatening gang members, and undeserving of human dignity.^14^ Although liberal arguments around helping asylum seekers on the basis of morality, inclusion, and compassion are important,^2^ the United States needs to take serious responsibilities for the current situation and make up for its well-documented history of meddling in Central America.^15^ Indeed, the United States has over a century-long history of political, military, and economical interventions in Central America and is basically reaping what it has sown^15^; actions that could be traced back to Theodore Roosevelt (ie, the 26th President of the United States) justifying the US’s right to act as an “international police power” in Latin America (1904),^16^ to the failed war on drugs policy in Latin America (1982),^17,18^ and the 2006 US-Central American Free Trade Agreement (CAFTA).^15,19^

These policies and military interventions throughout the decades (eg, backing up coup attempts), have left generational scars all over the socio-economic and political climate of the Northern Triangle; realities that are inseparable from the current humanitarian crisis of human movement and displacement in Central America.^15^ CAFTA for example, was portrayed as a free-trade panacea for the Central America’s refugee crisis, illicit drug trafficking, and gang violence.^20^ While CAFTA has had some positive economic outcomes for the countries involved, it has arguably caused more harms than good and served as a structural factor for displacing the rural population and taking the jobs of small family farmers who could not compete with duty-free and subsidized American grain products.^20-22^ The unemployment problem is most pronounced among young men in the Northern Triangle which would facilitate their recruitment by gangs and fuel the regional violence. Twelve years after CAFTA, Northern Triangle is now one of the most violent regions of the world.^12,20^

## What Are the Social and Health Costs?

The bitter irony of the crisis is that no one is going to benefit from the current situation in the Southern border of the United States. What is going on is a vicious humanitarian cycle that only serves the instability and gang violence in the region. While Mr. Trump is all about ‘winning,’^23^ what is happening at the US’s borders is nothing but a lose-lose situation both for the United States and the asylum seekers. Those asylum seekers whose application is rejected have to go back to their violent, threatening, and unstable environment. Conditions that would leave them with limited options that often lead to internal displacement, gang recruitments, or forcing them to take riskier paths to ‘safety’ as most do not have any safer alternatives.^24,25^

Conversely, those asylum seekers whose application is finally approved but have experienced long detentions and family separations are likely to become in need of the healthcare and social services in the short and long run.^26^ The story of the Honduran father who committed suicide after being separated from his child and wife speaks to the immediate distressing impacts of such senseless policies on families^27^; the long-term outcomes are even worse. Furthermore, the effects of separation from parents would be more devastating for children. Numerous studies have shown the adverse short-term, medium-term, and long-term impacts of childhood trauma and adverse childhood experiences among children on their physical and mental health throughout their life course.^6,28^ Several public health agencies (eg, American Academy of Pediatrics) have issued statements warning the adverse and traumatizing effects of early childhood experiences and toxic stress on refugee children’s brain architecture.^29,30^ Trump’s administration should also learn from Canada’s recent experience with separating Indigenous children from their parents in residential schools; a policy that has led to generational traumas among the Indigenous communities across Canada and significant long-term societal, cultural, and economic costs.^6,31-33^

All in all, choosing to ignore previous US governments’ actions in the humanitarian crisis in the Northern Triangle would only delay any practical solution to the crisis. Seeking refuge in the United States is deeply rooted in Central America’s social, economic, and political circumstances and the United States cannot incarcerate or criminalize its way out of it. Any sustainable and lasting response should consider the complex nature of the crisis and include not only military aids but also increased supports in development, anti-corruption efforts, healthcare, education, and employment opportunity generation. We are at a critical point in the contemporary history — What Mr. Trump and his administration should consider is how they will be judged by the history and whether they want to be on its right side.

## Acknowledgement

Mohammad Karamouzian is a PhD student at the University of British Columbia, Vancouver, BC, Canada who is supported by the Vanier Canada Graduate Scholarship and the Pierre Elliott Trudeau Foundation Doctoral Scholarships.

## Ethical issues

Not applicable.

## Competing interests

Author declares that he has no competing interests.

## Author’s contribution

MK is the single author of the paper.
